# Structured auscultation evidence modeling for multi-protocol respiratory sound classification

**DOI:** 10.3389/fmed.2026.1987793

**Published:** 2026-10-21

**Authors:** Yuan Yuan, Qi Zhang, Yinuo Liu, Yang Chen, Xiaowei Wang

**Affiliations:** 1 Department of Respiratory and Critical Care Medicine, Shanghai Changzheng Hospital, Naval Medical University, Shanghai, China; 2 Department of Bioinformatics, Center for Translational Medicine, Naval Medical University, Shanghai, China; 3 Department of Dermatology, Shanghai Changzheng Hospital, Naval Medical University, Shanghai, China; 4 Department of Thoracic Surgery, Shanghai Changhai Hospital, Naval Medical University, Shanghai, China

**Keywords:** artificial intelligence in healthcare, biomedical signal processing, biosignal classification, computer-assisted auscultation, digital auscultation, respiratory sound analysis

## Abstract

**Introduction:**

Digitally recorded respiratory sounds provide a practical source of physiological information for computer-assisted auscultation. Their acoustic presentation varies across subjects, recording locations, devices, filtering conditions, and environmental noise. Most classification models map acoustic representations directly to disease labels, which provides limited access to the auscultation findings associated with each prediction. This study investigated whether respiratory acoustic representations could be organized into structured evidence for classification across different label settings and evaluation levels.

**Methods:**

We developed *Evi-CARD* to organize respiratory sound representations into structured evidence for classification. A frozen Open Respiratory Acoustic Foundation Model (OPERA) encodes overlapping 8s segments, from which a concept-aligned pathway estimates wheeze, crackle, inspiration, and expiration together with learned evidence weights. An attention-constrained residual pathway preserves complementary acoustic information, although its attention ceiling does not strictly bound the residual pathway’s contribution to the final logits. Separate disease-query layers adapt the shared evidence representation to each classification setting. We assessed BRACETS A2 and A3 by subject-independent five-fold validation at the recording level and KAUH-primary by subject-independent five-fold validation at the patient level for Healthy vs. Obstructive classification. Each assessment was conducted within its source cohort and was not an external-validation experiment.

**Results:**

On BRACETS, *Evi-CARD* achieved balanced accuracies of 77.8% for the A2 three-class task and 70.2% for the more granular A3 five-class task. On KAUH-primary, pooled patient-level out-of-fold evaluation yielded an area under the receiver operating characteristic curve of 95.1%, a balanced accuracy of 88.0%, a Macro F1 of 88.1%, and an expected calibration error of 5.1%. Across HF_Lung_V1, SPRSound, and ICBHI, the complete concept layer also provided more accurate and better-calibrated concept predictions than the evaluated acoustic-probe and concept-readout baselines.

**Discussion:**

These findings show that protocol-specific respiratory sound classification can be based on concept-associated latent evidence while retaining a constrained route for information outside the predefined concept set. However, *Evi-CARD* is not a strict scalar concept bottleneck, and no clinical meaning is assigned to its residual-memory slots. The evidence is limited to within-cohort cross-validation; independent-cohort testing, device- and site-stratified evaluation, and clinician review remain necessary before considering clinical deployment.

## 1 Introduction

Respiratory diseases remain a major source of morbidity and require accessible methods for early assessment and continued evaluation (1, 2). Auscultation is widely used to examine respiratory function because it is non-invasive, inexpensive, and readily available in clinical practice. Respiratory sounds such as wheezes and crackles provide information about airflow limitation and changes in pulmonary mechanics (3, 4). Digital auscultation extends conventional examination by allowing respiratory sounds to be recorded, stored, and analyzed computationally, as shown in Figure 1a. This has created a practical basis for classifying respiratory disease categories from digital respiratory sounds and for examining the acoustic findings associated with these predictions.

**Figure 1:**
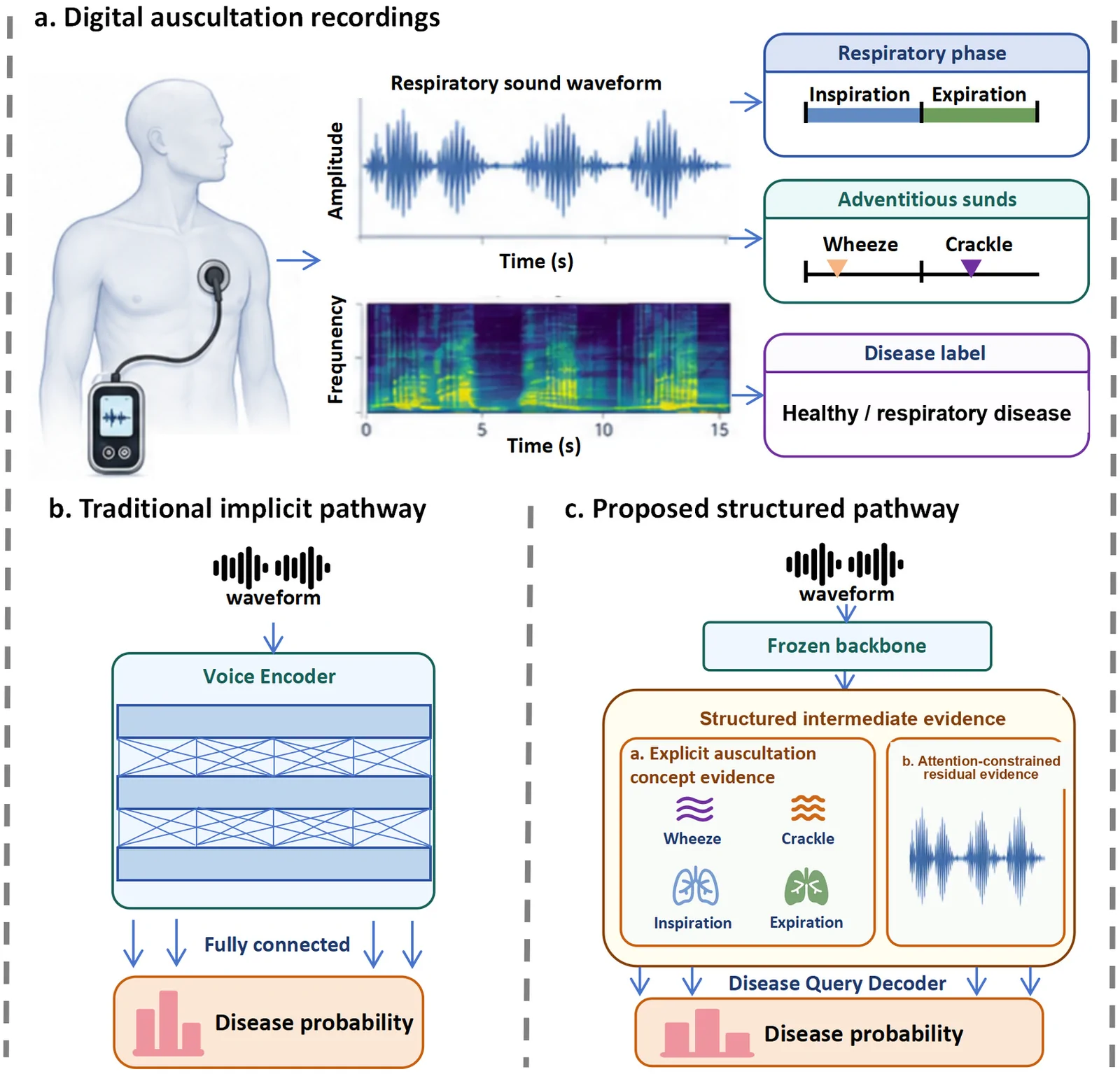
Digital auscultation and structured evidence modeling. **(a)** Digitally recorded respiratory sounds may be associated with respiratory-phase, adventitious-sound, and disease labels. **(b)** Conventional encoder-classifier pipelines generate disease probabilities from latent acoustic representations. This black-box mapping provides limited correspondence with the finding-based structure of clinical auscultation. **(c)** *Evi-CARD* organizes frozen-backbone representations into concept-aligned evidence for wheeze, crackle, inspiration, and expiration, while attention-constrained residual memory retains complementary information. The constraint limits disease-query attention allocated to residual positions; it does not impose a strict bound on their downstream logit contribution. A disease-query decoder integrates both evidence sources to produce protocol-specific disease probabilities.

Early computational studies relied on handcrafted descriptors, including mel-frequency cepstral coefficients, wavelet features, and spectral statistics, combined with task-specific classifiers (5–7). With the development of deep learning, convolutional and sequence-based models began to learn respiratory sound representations directly from spectrograms or audio recordings, reducing the dependence on manually designed features (7–9). More recently, transfer learning and respiratory acoustic foundation models have provided stronger representations for downstream tasks (10). These developments have enabled increasingly accurate classification of respiratory sounds across established benchmark settings.

Recent work has broadened respiratory-acoustic modeling while also exposing its deployment constraints. RespiraMFM combines respiratory sounds with clinical text across multiple datasets (11), and domain-adaptive learning has been evaluated for transferring electronic-stethoscope representations to smartphone recordings with separate-site validation (12). Studies conducted with noisy ICU recordings and real-world pediatric smartphone auscultation further show that acquisition environment, device characteristics, and population can materially affect performance (13, 14). Together with benchmarking of audio foundation models on heart and respiratory sounds (15), these findings indicate that model scale or within-dataset accuracy alone does not establish clinical generalization; subject-independent evaluation, calibration, acquisition context, and possible pretraining exposure must also be reported explicitly.

Despite these improvements, existing deep learning models often map acoustic representations directly to disease classes without showing which auscultatory findings contributed to the prediction, as shown in Figure 1b. This direct mapping makes it difficult to determine whether a model output is consistent with the respiratory signs present in the recording, limiting its transparency for clinical decision support (16, 17). Moreover, although saliency maps and attention visualizations can identify influential regions of an input (18, 19), these outputs are generally model-specific and do not directly represent clinically recognized respiratory sound characteristics. This motivates an intermediate representation that makes relevant auscultatory events and respiratory phases explicit within the classification process.

Concept-based learning offers a way to relate predictions to named attributes (20–22). Concept bottleneck and concept embedding models place these attributes directly within the prediction pathway (21, 23). Nevertheless, accurate concept recognition demonstrates alignment only at the predicted-concept level; it does not establish that every dimension of a learned concept representation is semantically pure or exclusively attributable to that concept. These approaches have also been studied in medical imaging, particularly in dermatology, where clinically recognized lesion characteristics are estimated and linked to skin disease predictions (24, 25). This provides a basis for making prediction-relevant findings explicit within a classification model. These studies in imaging provide inspiration for applying concept-based learning to respiratory sound analysis.

However, applying the concept-based learning to respiratory sounds directly introduces constraints. Wheezes and crackles vary over time and can exhibit different temporal and spectral forms (3, 26). Their presentation also varies with respiratory flow, body position, auscultation site, and recording conditions (4, 27). A further constraint is that wheeze, crackle, inspiration, and expiration capture only part of the acoustic variation associated with respiratory disease. Restricting classification to these four concepts could therefore discard informative patterns outside the predefined vocabulary. We address this incompleteness by pairing concept-aligned auscultation evidence with an attention-constrained residual pathway.

To address these issues, we propose *Evi-CARD*, a structured framework for respiratory sound classification. As shown in Figure 1c, it organizes respiratory acoustic tokens into concept-aligned streams for wheeze, crackle, inspiration, and expiration, with each stream combining a predicted intensity, a dictionary-guided direction, and a learned evidence weight. A constrained residual-memory pathway complements these streams with information not fully represented by the predefined concept vocabulary. Protocol-specific disease queries integrate both evidence sources to produce segment-level class probabilities. Because the decoder operates on rich latent concept and residual-memory vectors rather than scalar concepts alone, we do not present the architecture as a strict concept bottleneck.

We evaluated this shared evidence pathway under three distinct protocols: recording-level three-class classification on BRACETS A2, recording-level five-class classification on BRACETS A3, and patient-level Healthy vs. Obstructive classification on KAUH-primary. Concept and auxiliary disease supervision came from their designated source datasets, whereas each classification task was evaluated within its own cohort. The study therefore examines whether one pretrained evidence pathway can support different label definitions and evaluation units, but it does not test transfer across hospitals, devices, geographic populations, or prospective cohorts.

## 2 Materials and methods

### 2.1 Datasets and study design

The six public respiratory sound datasets served complementary roles. Table 1 summarizes their cohort and recording counts, labels used in this study, acquisition information, and protocol-specific inclusion. BRACETS supported recording-level disease classification (28), whereas KAUH-primary supported patient-level Healthy vs. Obstructive classification (9). HF_Lung_V1, SPRSound, and ICBHI contributed the available annotations for wheeze, crackle, inspiration, and expiration (8, 27, 29), and RespiratoryDatabase@TR supplied auxiliary COPD-related supervision during Stage 1 evidence-pathway training (30). Because the source annotations and intended uses differed, the datasets retained their original label structures rather than being merged into a common disease taxonomy.

**Table 1:** Characteristics and roles of the respiratory-sound datasets.

Dataset	Cohort/recordings	Labels used here	Acquisition information	Study role and inclusion
BRACETS	78 adult participants; 1,097 respiratory-sound recordings	A2: Healthy, Obstructive, Restrictive; A3: Healthy, COPD, Asthma, ILD, Pulmonary Infection	Multi-site digital auscultation; source-device and demographic details follow the original release	Recording-level downstream disease classification under separate subject-independent within-cohort A2 and A3 protocols
KAUH	112 participants; one recording per participant	Healthy, Asthma, COPD, and other respiratory/cardiac conditions in the source dataset	3M Littmann 3200; Bell, Diaphragm, and Extended filtered views; source sampling protocol retained before resampling	Patient-level downstream disease evaluation. KAUH-primary includes Healthy (35), Asthma (32), and COPD (9); Asthma and COPD form the Obstructive class, and the remaining 36 cases are excluded from this binary protocol
HF_Lung_V1	261 participants; 9,765 15 s recordings	Wheeze, crackle, inspiration, and expiration when annotated	4 kHz; Littmann 3200 and HF-Type-1 devices	Concept supervision and fold-held-out downstream concept evaluation; possible source-level exposure during OPERA self-supervised pretraining
SPRSound	292 pediatric participants; 2,683 recordings and 9,089 annotated events	Adventitious sounds and respiratory phases	Digital-stethoscope recordings; source-native audio is resampled to 16 kHz	Concept supervision and fold-held-out downstream concept evaluation; no reported source-level exposure in the selected OPERA checkpoint and therefore used as a pretraining-exposure sensitivity setting
ICBHI	126 participants; 920 recordings and 6,898 respiratory cycles	Wheeze and crackle/cycle annotations	Multiple acquisition devices and sampling rates; resampled to 16 kHz	Concept supervision and fold-held-out downstream concept evaluation; possible source-level exposure during OPERA self-supervised pretraining
Respiratory-Database@TR	Subject-level multichannel recordings	COPD-related disease/severity labels	Multichannel respiratory acquisition; source-native audio is resampled to 16 kHz	Auxiliary COPD-related supervision during Stage 1 evidence-pathway training

Counts and demographic or device fields are reported only when available from the original dataset descriptions. OPERA is used as a frozen pretrained acoustic backbone in the present study. Possible source-level exposure during OPERA self-supervised pretraining therefore does not imply prior training on the downstream concept-recognition or disease-classification tasks evaluated here. All trainable downstream components are fitted within the corresponding supervised training folds. Post-segmentation clip counts and concept-positive/negative counts vary with segmentation and annotation overlap. Because fixed global values were not available in the source releases used here, they are not inferred retrospectively; this absence is stated explicitly rather than deferred to an unspecified future manifest.

Concept labels were harmonized according to the annotations available in each dataset. Continuous adventitious sounds were mapped to wheeze, whereas discontinuous adventitious sounds and coarse or fine crackles were mapped to crackle. Inspiration and expiration were used only when explicit respiratory-phase annotations were available. A concept was marked as present in an 8s clip when its annotated interval overlapped with the clip. Missing concept labels were masked during training and evaluation and were not treated as negative labels.

Concept-level performance was estimated from five-fold held-out predictions on HF_Lung_V1, SPRSound, and ICBHI. For each dataset and fold, all trainable concept-path components—including the adapters, projection layers, concept queries, dictionary prototypes, and associated evidence-pathway parameters—were fitted using only the other four folds; the held-out fold was excluded from fitting, early stopping, and parameter selection. Dataset-specific macro F1 scores included only the concepts annotated in that dataset, and respiratory-phase metrics included only samples with explicit inspiration or expiration labels. This separation prevents leakage during downstream supervised fitting but cannot undo possible exposure during OPERA’s self-supervised pretraining: HF_Lung_V1 and ICBHI may have contributed to that pretraining, whereas SPRSound was absent from the resources reported for the selected checkpoint and therefore serves as a source-level exposure sensitivity setting.

Disease classification followed the label definitions of each evaluation protocol. BRACETS A2 groups Asthma and COPD as Obstructive and ILD and Pulmonary Infection as Restrictive, yielding the three categories Healthy, Obstructive, and Restrictive. A3 retains the five original categories: Healthy, COPD, Asthma, ILD, and Pulmonary Infection. The source KAUH dataset contains 112 participants, of whom KAUH-primary retains 35 Healthy, 32 Asthma, and 9 COPD participants. Grouping Asthma and COPD as Obstructive yields 76 eligible participants (35 Healthy and 41 Obstructive); the remaining 36 participants with other respiratory or cardiac conditions are excluded from this binary protocol.

We constructed stratified, subject-independent five-fold outer splits before segmentation so that every recording and derived clip from a participant remained in the same fold. Within each outer-training partition, participants were divided into inner-training and validation subsets using a fixed random seed of 42 and an inner-validation proportion of 20% of each outer-training partition. The inner-training subset was used for model fitting, whereas the corresponding validation subset alone determined early stopping, hyperparameters, decision thresholds, and temperature scaling. The selected model and calibration parameters were then applied once to the held-out outer fold. Segment logits were aggregated within each original recording for BRACETS and across all recordings from each patient for KAUH-primary, matching the respective recording- and patient-level evaluation units. Stage 1 pretraining excluded KAUH-primary, and the resulting evidence pathway remained frozen during protocol-specific disease training.

### 2.2 Evi-CARD framework

*Evi-CARD* processes each recording as overlapping 8 s segments. OPERA first encodes each segment; the evidence pathway then extracts concept-aligned representations for wheeze, crackle, inspiration, and expiration, estimates their intensities and learned evidence weights, and retains complementary information through attention-constrained residual memory. Protocol-specific disease queries integrate these evidence sources to produce segment-level class probabilities.

#### 2.2.1 Acoustic representation and concept extraction.

The acoustic encoder converts each standardized respiratory sound segment into a representation that can support the four auscultation concepts. Figure 2 provides an overview of the framework. Each recording is standardized to 16kHz mono and segmented into 8s clips with a 4s stride in the main experiments. The encoder uses OPERA as the acoustic backbone (10), with lightweight bottleneck adapters inserted into selected middle-to-high layers. We used the publicly released OPERA-GT checkpoint (ViT-small, patch size 4, depth 12). Resampled 16 kHz audio was converted to a 64-bin log-mel spectrogram using a 64 ms window and a 32-ms frame shift. Along the time axis, each token advances by four frames, giving a 128 ms token stride; this value is the displacement between adjacent tokens, not the waveform span of one token. The four overlapping analysis windows cover approximately 64+3×32=160ms of waveform time. Because the standard ViT backbone preserves sequence length, the 128 ms stride and approximately 160 ms waveform coverage are unchanged across selected layers 6, 8, 10, and 12. Given a respiratory sound segment x, the encoder forms a multi-layer acoustic representation H. For the l-th selected layer, the adapted token representation is $H^{\prime \left(l\right)}\in R^{T_{l}\times d_{l}}$, where a token is one time-indexed encoder feature, $T_{l}$ is the number of tokens, and $d_{l}$ is the feature dimension.

**Figure 2:**
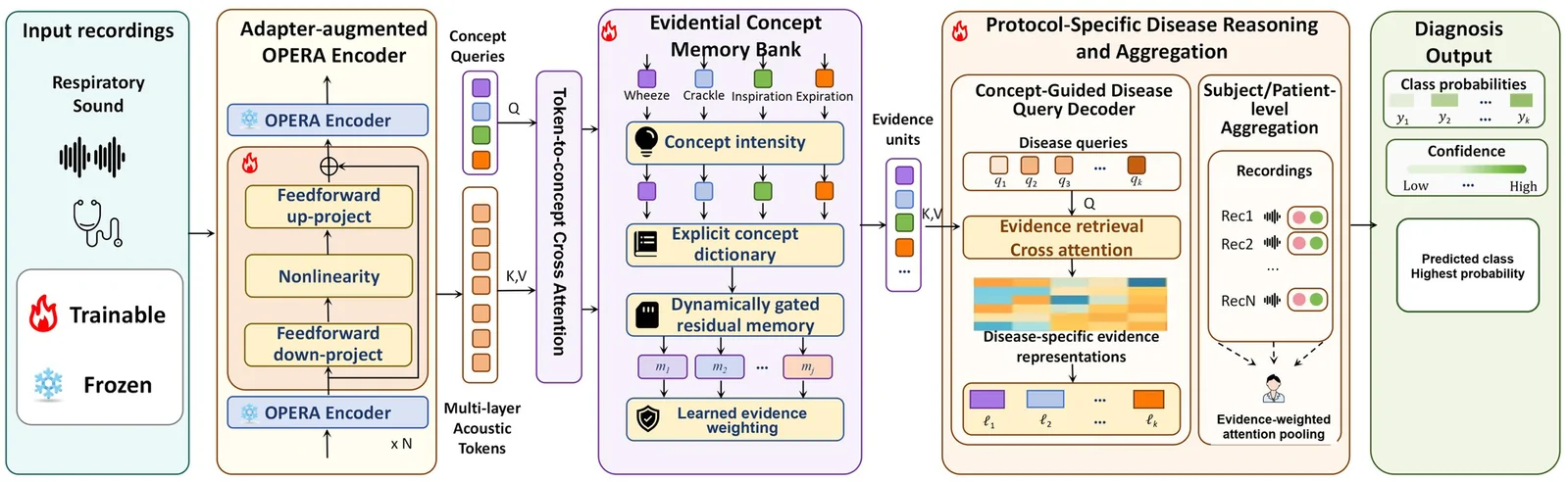
Overview of the proposed *Evi-CARD* framework. Respiratory sounds are encoded by the adapter-augmented OPERA acoustic encoder and transformed into structured auscultation evidence by the *Evidential Concept Memory Bank*. Its learned evidence weights are model-internal gates rather than physical reliability estimates. The *Concept-Guided Disease Query Decoder* performs protocol-specific disease reasoning over the evidence units, followed by evidence-weighted aggregation at the recording or patient level.

Respiratory phases and adventitious sounds are expressed through acoustic patterns with different temporal and spectral characteristics. Their representations may therefore be distributed across different encoder depths rather than concentrated in the final OPERA layer. Tokens from selected layers are combined so that each concept query can read from both intermediate and higher-level acoustic features. We design *Evidential Concept Memory Bank (ECMB)* to receive the multi-layer adapted token set. Each layer-specific token sequence is projected into a common evidence embedding space with dimension $d_{e}$ and concatenated into $\bar{H}$. The projection matrix is $W_{h}^{\left(l\right)}\in R^{d_{l}\times d_{e}}$ for layer l. Thus, $\bar{H}\in R^{(}\sum_{l\in L}T_{l})\times d_{e}$ provides the acoustic basis for concept extraction, where L denotes the selected OPERA layers.

The concept set comprises wheeze, crackle, inspiration, and expiration. Wheeze and crackle represent adventitious sound evidence, while inspiration and expiration provide respiratory-phase evidence. These four concept streams are retained separately before they are combined into structured evidence. For each concept c∈C, *ECMB* maintains a learnable concept query $q_{c}\in R^{d_{e}}$. A query is a learnable vector that reads evidence for one target concept from the acoustic representation. The *Multi-layer Query (MLQ)* mechanism applies multi-head cross-attention to obtain a concept-specific acoustic token and its intensity, as defined in Equation 1:

$$\begin{array}{cc} z_{c} & =\text{MHA}(q_{c},\bar{H},\bar{H}),\\ s_{c} & =\sigma ({\text{MLP}}_{s}(z_{c})),\;c\in C. \end{array}$$ (1)

Here, $z_{c}\in R^{d_{e}}$ is the acoustic concept token for concept c, and $s_{c}\in [0,1]$ is its predicted intensity. When concept labels are available, a masked concept-prediction loss is used, where ${\hat{s}}_{c}$ is the concept label and $\eta_{c}\in \{0,1\}$ indicates whether the label is available for the current sample. The mask permits training with partially annotated concept labels.

The same concept can have different acoustic forms. *ECMB* therefore maintains an explicit concept dictionary with K learnable prototypes $\{p_{c,k}{\}}_{k=1}^{K}$ for each concept, where $p_{c,k}\in R^{d_{e}}$. A prototype is a learned representative direction for one acoustic form of a concept. Temperature-scaled cosine similarity produces the matching weights and the dictionary-guided concept direction, as defined in Equation 2:

$$\begin{array}{cc} \alpha_{c,k} & =\frac{\exp\;(\cos\;(z_{c},p_{c,k})/\tau )}{\sum_{k^{\prime }=1}^{K}\exp\;(\cos\;(z_{c},p_{c,k^{\prime }})/\tau )},\\ d_{c} & =\sum_{k=1}^{K}\alpha_{c,k}p_{c,k}. \end{array}$$ (2)

Here, τ is a temperature parameter. The variables $z_{c}$, $s_{c}$, and $d_{c}$ denote the acoustic concept token, concept intensity, and dictionary-guided concept direction, respectively.

#### 2.2.2 Concept-aligned and residual evidence construction

For each concept, the model combines its acoustic token, predicted intensity, and dictionary-guided direction into a concept-aligned evidence unit. *ECMB* also predicts an evidence weight $r_{c}\in [0,1]$, as defined in Equation 3:

$${\tilde{e}}_{c}=r_{c}\text{LN}\left(W_{e}[z_{c};d_{c}]+w_{s}s_{c}\right),$$ (3)

where LN(⋅) denotes layer normalization, $W_{e}$ is a learnable projection, $w_{s}\in R^{d_{e}}$ maps the scalar intensity into the evidence space, and [⋅;⋅] denotes concatenation. The set $E_{c}=\{{\tilde{e}}_{c}{\}}_{c\in C}$ forms the concept-aligned portion of the structured evidence, retaining the concept-specific acoustic feature and its dictionary-guided direction for use by the decoder. Concept and memory weights are learned end-to-end through the Stage 1 objectives without separate reliability labels. They are therefore model-internal gates, not validated measurements of signal quality, recording fidelity, environmental noise, or annotator agreement.

To retain acoustic variation not fully represented by the predefined vocabulary, *ECMB* derives a residual representation from the discrepancies between the acoustic concept tokens $z_{c}$ and their dictionary-guided directions $d_{c}$. Dynamically gated memory then converts this representation into residual evidence, as defined in Equation 4:

$$\begin{array}{cc} u & ={\mathrm{MLP}}_{\mathrm{res}}\;({\mathrm{Concat}}_{c\in C}\;[z_{c}-d_{c}]),\\ {\tilde{e}}_{j}^{m} & =r_{j}^{m}g_{j}m_{j},\;j=1,\ldots ,J. \end{array}$$ (4)

Here, $u\in R^{d_{e}}$ is the residual representation and $\{m_{\;j}{\}}_{\;j=1}^{J}$ are J learnable memory slots in $R^{d_{e}}$. For slot j, $g_{j}\in [0,1]$ denotes its gated activation and $r_{j}^{m}\in [0,1]$ is a learned weight estimated from $\left[u;m_{j};g_{j}\right]$; their product with $m_{j}$ yields the weighted residual-memory evidence unit ${\tilde{e}}_{\;j}^{m}$. Although the residual is derived from concept-associated tokens, it may encode information outside the four named concepts and is not assigned a clinical interpretation.

A sparsity penalty limits the number of memory slots activated by each sample, while an orthogonality penalty discourages those slots from duplicating concept-aligned directions. The resulting concept-aligned and residual evidence units form the evidence matrix E. For downstream processing, *ECMB* also returns the concept intensities s, concept weights $r^{c}$, memory weights $r^{m}$, and memory activations g.

#### 2.2.3 Protocol-specific disease classification

The *Concept-Guided Disease Query Decoder (CDQD)* maps the evidence matrix to the category set $Y^{q}$ for diagnostic protocol q. It maintains a learnable disease query $q_{y}^{q}\in R^{d_{e}}$ for each category $y\in Y^{q}$. Each query reads the relevant evidence through multi-head cross-attention and produces a logit $\ell_{y}^{q}$ and probability $p_{y}^{q}$:

$$\begin{array}{cc} o_{y}^{q} & =\mathrm{MHA}\;(q_{y}^{q},E,E),\\ \ell_{y}^{q} & ={\mathrm{MLP}}_{q}\;(o_{y}^{q}),\;p_{y}^{q}=\frac{\exp\;(\ell_{y}^{q})}{\sum_{y^{\prime }\in Y^{q}}\exp\;(\ell_{y^{\prime }}^{q})},\;y\in Y^{q}. \end{array}$$ (5)

Here, $o_{y}^{q}$ is the disease-specific evidence representation. The keys and values of *CDQD* are restricted to E, so each category reads from the same evidence matrix through its own query. The resulting segment-level logits are passed to segment aggregation.

The total disease-query attention assigned to memory evidence is capped. Let $A_{y,j}^{m}$ denote the attention weight from disease query y to memory evidence unit ${\tilde{e}}_{\;j}^{m}$, obtained from the multi-head cross-attention in Equation 5 by averaging attention scores across all heads for the memory positions. The memory attention is limited by a prescribed ceiling:

$${\hat{A}}_{y,j}^{m}=\gamma_{y}A_{y,j}^{m},\;\gamma_{y}=\min\left(1,\frac{\rho }{\sum_{\;j=1}^{J}A_{y,j}^{m}+\epsilon }\right).$$ (6)

Here, ϵ provides numerical stability. After applying the cap, the remaining attention mass is allocated to the concept-aligned evidence and all weights are renormalized before recomputing $o_{y}^{q}$. Equation 6 therefore constrains attention allocation to residual-memory positions, but it cannot impose a strict bound on their causal or logit-level contribution after value projection and subsequent transformations. We accordingly refer to the mechanism as bounded residual-memory attention allocation, not bounded functional contribution.

#### 2.2.4 Segment aggregation

*Evi-CARD* produces one logit vector for each 8s segment. For BRACETS, segment logits from the same original recording are aggregated into one recording-level prediction. For KAUH-primary, segment logits from all recordings of the same patient are jointly aggregated into one patient-level prediction. The original recording and the patient are therefore the evaluation units for BRACETS and KAUH-primary, respectively.

Let $I_{z}$ denote the respiratory sound segments belonging to evaluation unit z, where z=r is an original BRACETS recording and z=u is a KAUH-primary patient. For segment $i\in I_{z}$ under protocol q, *CDQD* produces the class-logit vector $\ell_{i}^{q}$ and *ECMB* provides the concept weights $\{r_{i,c}{\}}_{c\in C}$. Their mean, ${\bar{r}}_{i}$, summarizes segment-level concept evidence. A protocol-specific attention scorer uses $\ell_{i}^{q}$ and ${\bar{r}}_{i}$ to assign the normalized aggregation weight $a_{i}$, yielding

$$\ell_{z}^{q}=\sum_{i\in I_{z}}a_{i}\ell_{i}^{q},\;p_{z}^{q}=\text{softmax}\left(\ell_{z}^{q}\right).$$ (7)

Here, $\ell_{z}^{q}$ and $p_{z}^{q}$ are the final logit and probability vectors for the evaluation unit.

### 2.3 Experimental setup and evaluation protocol

Training is divided into four stages to separate data partitioning, shared evidence learning, and protocol-specific adaptation. Stage 0 prepares subject-independent partitions and acoustic inputs. Stage 1 learns the shared evidence pathway from concept labels and source-side auxiliary disease supervision. With this pathway fixed, Stage 2 fits the complete disease-query module for each protocol. Stage 3 initializes from the Stage 2 solution and uses a lower learning rate to refine the disease-query embeddings and output layer together with fold-specific calibration. This design prevents protocol-specific disease labels from continuously reshaping the shared concept-aligned representation.

Stage 0 comprises manifest generation, subject-independent fold assignment, audio standardization, segmentation, and feature caching. Fold assignment precedes segmentation, ensuring that overlapping clips from a participant cannot cross training, validation, and test partitions. Recordings are then resampled to 16kHz mono and divided into 8s clips with a 4s stride before OPERA preprocessing.

Stage 1 draws concept supervision from HF_Lung_V1, SPRSound, and ICBHI and auxiliary COPD-related supervision from RespiratoryDatabase@TR, while keeping OPERA frozen. Lightweight adapters with bottleneck dimension r=32 are inserted at layers L={6,8,10,12}. The evidence space has dimension $d_{e}=256$, and the attention modules use four heads with dropout 0.10. Each concept dictionary contains K=8 prototypes with cosine-similarity temperature 0.07; the residual memory likewise contains J=8 slots, with residual attention capped at ρ=0.35. We set $\lambda_{\mathrm{con}}=1.00$, $\lambda_{\mathrm{sparse}}=1.0\times 10^{-3}$, and $\lambda_{\mathrm{orth}}=1.0\times 10^{-2}$. Optimization used AdamW with weight decay $1.0\times 10^{-4}$, a learning rate of $1.0\times 10^{-3}$, at most 50 epochs, a batch size of 32, and a fixed random seed of 42. We used default PyTorch initialization, without class weighting, oversampling, or audio augmentation beyond resampling and segmentation. For each supervised concept fold, all trainable evidence-pathway components, including the prototypes, were reinitialized and fitted exclusively on the corresponding training partition.

Stage 2 fits a protocol-specific *CDQD* while the evidence pathway remains fixed. BRACETS A2 and A3 supervise their respective multiclass disease queries and output MLPs, whereas KAUH-primary supervises a binary counterpart. Before loss calculation, segment logits are aggregated at the recording level for BRACETS and at the patient level for KAUH-primary. Training uses AdamW with a learning rate of $1.0\times 10^{-3}$, weight decay $1.0\times 10^{-4}$, a batch size of 32, and at most 50 epochs; the Brier-loss weight is $\lambda_{\mathrm{cal}}=0.10$.

Stage 3 refines only the disease-query embeddings and output MLP within each outer-training partition, initializing from the fitted Stage 2 *CDQD*; temperature scaling begins at T=1.0. AdamW optimization uses a learning rate of $5.0\times 10^{-4}$, weight decay $1.0\times 10^{-4}$, a batch size of 32, and at most 20 epochs. Early stopping is based on inner-validation balanced accuracy with patience 10. The selected checkpoint, decision threshold, and temperature are then applied once to the held-out outer fold. Held-out KAUH-primary predictions are retained at the patient level; pooled and fold-summary estimands are reported separately as defined below.

The principal experiments use random seed 42. For the three-seed robustness analysis, each evaluated configuration was reinitialized and refitted with seeds {42,43,44}, while the subject-independent outer folds and their inner-training/validation membership were held fixed. This design measures optimization variability without conflating it with a different data partition.

Disease supervision uses cross-entropy loss $L_{\mathrm{cls}}$ on aggregated predictions. A standard multiclass Brier loss $L_{\mathrm{Brier}}$ is also computed between the aggregated probability vector and the one-hot label of each evaluation unit. The overall objective is defined in Equation 8:

$$L=L_{\mathrm{cls}}+\lambda_{\mathrm{con}}L_{\mathrm{con}}+\lambda_{\mathrm{sparse}}L_{\mathrm{sparse}}+\lambda_{\mathrm{orth}}L_{\mathrm{orth}}+\lambda_{\mathrm{cal}}L_{\mathrm{Brier}}.$$ (8)

During Stage 1, $L_{\mathrm{cls}}$ denotes the auxiliary COPD-related classification loss computed on RespiratoryDatabase@TR. During Stages 2 and 3, it denotes the protocol-specific classification loss computed from aggregated BRACETS or KAUH-primary predictions. The auxiliary classification head used in Stage 1 is discarded after evidence-pathway pretraining. Here, $\lambda_{\mathrm{con}}$, $\lambda_{\mathrm{sparse}}$, $\lambda_{\mathrm{orth}}$, and $\lambda_{\mathrm{cal}}$ are weighting coefficients. The concept loss is applied to samples with concept annotations. Auxiliary disease supervision is used only during evidence-pathway pretraining. The disease classification and Brier losses are applied to aggregated predictions from the training partition of each protocol, at the recording level for BRACETS and at the patient level for KAUH-primary.

The *Concept Bottleneck* baseline (21) shares the OPERA backbone, adapter configuration, concept vocabulary, Stage 1 supervision, and protocol-specific optimization schedule used by *Evi-CARD*. Its adapters use bottleneck dimension r=32 at layers L={6,8,10,12}, and its concept set is C={wheeze,crackle,inspiration,expiration}. A single-layer linear projection with sigmoid activation predicts the concept-intensity vector $s=[s_{c}{]}_{c\in C}$ from final-layer OPERA tokens, after which a linear classifier produces dataset-specific disease predictions. For comparability, the baseline uses the aggregation rule in Equation 7 with a uniform evidence-weight proxy ${\bar{r}}_{i}=1$. Thus, it shares the data and training controls of *Evi-CARD* but restricts the disease readout to scalar concept intensities and omits MLQ, the explicit concept dictionary, gated residual memory, learned evidence weighting, and the *CDQD* attention decoder.

All baseline results in Tables 2, 3 come from our reimplementations under the same subject-independent splits, aggregation rules, and calibration pipeline; none were transcribed from prior publications. This common evaluation protocol also permits fold-wise standard deviations, Brier scores, and ECE to be reported for every baseline.

**Table 2:** BRACETS primary recording-level multi-class evaluation results across A2 (three-class) and A3 (five-class) settings.

Method	AUROC	AUPRC	BAcc	Macro	Disease	Healthy	Brier	ECE
	(%)	(%)	(%)	F1 (%)	recall (%)	recall (%)	↓	(%) ↓
A2: Three-class protocol (Healthy, Obstructive, Restrictive)								
AST	85.6 (85.2 ± 2.9)	74.1 (73.4 ± 4.1)	63.4 (62.8 ± 3.7)	62.2 (61.9 ± 4.4)	70.8 (70.1 ± 3.5)	48.6 (48.2 ± 6.8)	0.41 (0.42 ± 0.04)	19.2 (18.9 ± 2.2)
CLAP linear probe	88.1 (87.7 ± 1.8)	77.5 (76.9 ± 3.6)	67.2 (66.8 ± 4.1)	66.4 (65.9 ± 3.6)	74.2 (73.8 ± 3.1)	53.1 (52.7 ± 5.9)	0.37 (0.38 ± 0.03)	17.6 (17.9 ± 1.8)
OPERA linear probe	89.7 (89.4 ± 2.1)	79.4 (79.0 ± 2.7)	69.3 (69.0 ± 3.0)	68.5 (68.2 ± 3.3)	78.0 (77.5 ± 2.8)	51.9 (52.1 ± 5.2)	0.34 (0.35 ± 0.03)	16.8 (16.4 ± 1.6)
OPERA adapter	89.2 (89.0 ± 1.5)	80.8 (80.2 ± 2.9)	72.0 (71.5 ± 2.7)	71.6 (71.0 ± 3.1)	80.7 (80.1 ± 2.4)	54.5 (54.4 ± 4.7)	0.33 (0.33 ± 0.02)	15.1 (15.4 ± 1.7)
Evi-CARD with frozen evidence extractor	91.4 (91.0 ± 1.3)	82.1 (81.8 ± 2.0)	74.7 (74.4 ± 2.4)	74.3 (74.0 ± 2.1)	83.5 (83.2 ± 2.0)	57.2 (56.8 ± 4.1)	0.30 (0.31 ± 0.03)	14.0 (13.7 ± 1.3)
Evi-CARD with query tuning	**92.7** **(92.6 ± 0.7)**	**84.0** **(84.0 ± 1.4)**	**77.8** **(77.8 ± 1.3)**	**77.5** **(77.5 ± 1.4)**	**87.7** **(87.7 ± 1.1)**	**58.0** **(58.0 ± 3.6)**	**0.28** **(0.28 ± 0.02)**	**13.1** **(13.1 ± 1.1)**
A3: Five-class protocol (Healthy, COPD, Asthma, ILD, Pulmonary Infection)								
AST	79.1 (78.7 ± 3.2)	63.4 (62.8 ± 4.3)	52.7 (52.3 ± 4.5)	51.7 (51.2 ± 4.7)	51.6 (51.2 ± 4.3)	57.2 (56.6 ± 7.4)	0.59 (0.60 ± 0.05)	24.6 (24.2 ± 2.6)
CLAP linear probe	82.3 (82.5 ± 2.7)	66.8 (66.2 ± 3.9)	56.0 (55.5 ± 4.1)	55.4 (54.9 ± 4.3)	56.2 (55.7 ± 3.9)	55.1 (54.8 ± 6.7)	0.56 (0.57 ± 0.04)	23.1 (23.4 ± 2.1)
OPERA linear probe	85.2 (84.8 ± 2.5)	70.1 (69.6 ± 3.2)	60.2 (59.7 ± 3.6)	59.1 (58.8 ± 3.9)	60.0 (59.5 ± 3.4)	61.0 (60.4 ± 5.8)	0.52 (0.53 ± 0.04)	21.2 (20.9 ± 1.9)
OPERA adapter	84.7 (84.9 ± 1.8)	71.9 (71.3 ± 2.9)	63.3 (62.8 ± 3.0)	62.9 (62.3 ± 3.4)	64.2 (63.7±2.8)	59.8 (59.3 ± 5.2)	0.50 (0.49 ± 0.03)	20.4 (20.1 ± 1.8)
Evi-CARD with frozen evidence extractor	89.8 (89.3 ± 1.5)	74.5 (74.0 ± 2.3)	67.3 (66.9 ± 2.6)	66.5 (66.0±2.8)	68.1 (67.7±2.4)	64.2 (63.5 ± 4.5)	0.47 (0.48 ± 0.03)	18.9 (19.2 ± 1.4)
Evi-CARD with query tuning	**91.8** **(91.9 ± 0.7)**	**77.3** **(77.3±1.7)**	**70.2** **(70.3±1.7)**	**69.1** **(69.1 ± 1.6)**	**71.1** **(71.2 ± 1.8)**	**66.5** **(66.6 ± 3.7)**	**0.45** **(0.45 ± 0.02)**	**18.0** **(18.0 ± 1.2)**

BAcc denotes balanced accuracy. Disease Recall is macro-average recall over the non-Healthy classes, and Healthy Recall is recall for the Healthy class. The first line gives the pooled point estimate; parenthetical values give the unweighted outer-fold mean ± standard deviation. Accordingly, the pooled Macro AUROCs are 92.7% (0.927) for A2 and 91.8% (0.918) for A3, matching Figure 3; the parenthetical 92.6 ± 0.7% and 91.9 ± 0.7% values are fold summaries rather than alternative pooled estimates. The pooled A2/A3 threshold-dependent values are calculated from the exact integer counts in Figure 4.

Bold values indicate the best performance among the evaluated methods for each metric within the corresponding classification protocol. For AUROC, AUPRC, BAcc, Macro F1, Disease Recall, and Healthy Recall, higher values indicate better performance; for Brier score and ECE, lower values indicate better performance.

**Table 3:** KAUH-primary five-fold patient-level validation performance.

Method	AUROC	AUPRC	BAcc	Macro	Sens.	Spec.	Brier	ECE
	(%)	(%)	(%)	F1 (%)	(%)	(%)	↓	(%) ↓
ResNet50 (33)	80.2 (80.1 ± 5.2)	78.4 (78.7 ± 3.8)	71.6 (71.4 ± 8.4)	70.8 (70.9 ± 5.5)	71.2 (71.0 ± 6.3)	72.0 (71.8 ± 13.7)	0.323 (0.324 ± 0.031)	18.4 (18.6 ± 5.1)
AST (31)	82.7 (82.8 ± 4.1)	80.6 (80.7 ± 3.5)	73.9 (73.9±6.1)	72.4 (72.7±7.8)	73.0 (73.2±8.2)	74.8 (74.6 ± 11.8)	0.297 (0.299 ± 0.028)	16.2 (16.0±4.7)
MTL-MobileNet (34)	89.1 (88.8 ± 3.7)	87.5 (87.6 ± 4.2)	82.2 (82.0±7.5)	80.8 (81.1±5.9)	81.0 (80.8 ± 5.1)	83.4 (83.2 ± 10.4)	0.221 (0.219 ± 0.025)	11.6 (11.4 ± 3.9)
CLAP linear probe (32)	84.6 (84.8 ± 4.7)	82.8 (82.6 ± 3.1)	75.8 (75.8±6.8)	74.2 (73.9±6.4)	74.5 (74.6 ± 7.7)	77.1 (76.9 ± 12.3)	0.267 (0.269 ± 0.034)	14.3 (14.6 ± 4.3)
HeAR linear probe (35)	86.3 (86.6 ± 3.3)	84.5 (84.7 ± 2.9)	77.4 (77.4±7.2)	75.8 (75.5±4.8)	76.0 (76.1 ± 5.7)	78.8 (78.6 ± 11.2)	0.244 (0.239 ± 0.027)	13.1 (13.0 ± 4.6)
OPERA linear probe (10)	92.4 (92.7 ± 3.8)	91.1 (90.9 ± 2.6)	85.8 (85.9±5.8)	84.2 (84.1±6.1)	84.5 (84.6 ± 7.0)	87.1 (87.2 ± 9.3)	0.197 (0.204 ± 0.022)	9.8 (10.0 ± 3.5)
OPERA adapter (36)	93.6 (93.9 ± 2.9)	92.5 (92.8 ± 3.2)	87.4 (87.3±5.3)	86.5 (86.8±5.7)	86.8 (86.5 ± 6.4)	88.0 (88.1 ± 8.6)	0.101 (0.103 ± 0.019)	6.4 (6.3 ± 3.1)
Concept bottleneck (21)	92.9 (92.8 ± 3.6)	91.8 (91.7 ± 2.4)	86.0 (85.9±5.0)	85.2 (85.1±6.8)	85.5 (85.4 ± 5.5)	86.5 (86.3 ± 9.1)	0.118 (0.113 ± 0.024)	7.1 (7.3 ± 3.8)
Evi-CARD	94.4 (94.1 ±3.1)	93.4 (93.7± 2.8)	88.8 (88.8 ± 5.6)	88.0 (87.8±4.5)	88.3 (88.0±5.9)	89.3 (89.5 ± 8.0)	0.086 (0.088 ± 0.013)	5.3 (5.4 ± 2.9)
Evi-CARD with KAUH query tuning	95.1 (95.3 ± 2.7)	93.8 (93.5 ± 3.5)	88.0 (89.2±4.8)	88.1 (88.2±5.2)	90.2 (90.3 ± 5.3)	85.7 (88.0 ± 7.5)	0.083 (0.081 ± 0.017)	5.1 (4.8 ± 3.3)

Results on KAUH-primary five-fold patient-level validation. BAcc denotes balanced accuracy. Sensitivity and specificity are computed for the Obstructive class. The first line gives the pooled out-of-fold point estimate over 76 patients; parenthetical values give the unweighted outer-fold mean ± standard deviation. The pooled balanced accuracy (88.0%) and Macro F1 (88.1%) are the point estimates paired with the bootstrap intervals in Section 3.3.

All experiments used PyTorch 2.2.1 and a single NVIDIA RTX 4090 GPU, with OPERA instantiated from its public implementation. Disease-level evaluation covered AUROC, AUPRC, balanced accuracy, macro F1, sensitivity, specificity, Brier score, and ECE. Concept-level evaluation covered per-concept F1 and AUPRC, macro F1, mean average precision, and C-ECE.

Global disease-level ECE and Brier scores were computed after fold-specific temperature scaling. ECE used 10 equal-width bins on [0,1]; for multiclass predictions, confidence was the maximum class probability and accuracy indicated whether the predicted class was correct. Each nonempty bin contributed its absolute confidence–accuracy gap weighted by its share of evaluation units, while empty bins received zero weight. The reviewer-requested post hoc KAUH-primary analysis used the archived pooled out-of-fold probabilities without an additional temperature transformation in the analysis script. Classwise ECE was computed separately from the Obstructive probability and its complementary Healthy probability against the corresponding one-vs-rest labels, and intermediate-range ECE retained only patients with an Obstructive probability in [0.20,0.80]. Classwise Brier scores were reported as the algebraic one-vs-rest decomposition of the global binary score. C-ECE applied the same 10-bin calculation to each available binary concept probability and then averaged across concepts. For multiclass BRACETS predictions, the Brier score was the mean summed squared error over the aggregated class-probability vector. For binary KAUH-primary predictions, it was the mean squared error of the Obstructive probability, which equals the average of the two one-vs-rest Brier components.

BRACETS and KAUH-primary point estimates, bootstrap intervals, and calibration analyses were computed from pooled out-of-fold predictions after recording-level aggregation for BRACETS and patient-level aggregation for KAUH-primary; each evaluation unit therefore contributed once. In Tables 2, 3, the first line reports this pooled point estimate, whereas parenthetical values report the unweighted mean and standard deviation across the five outer folds. The pooled point estimate is the reference value used in the Abstract, Results, and bootstrap analysis. Within each fold, the decision threshold and temperature were selected solely from the corresponding inner-training and validation data; AUROC and AUPRC remained threshold-independent.

For visualization only, t-SNE was applied to standardized centered-log-ratio transforms of the full class-probability vectors. We used two output dimensions, Euclidean distance, PCA initialization, an automatic learning rate, early exaggeration of 6, 2,000 iterations, and random seed 42. Perplexity was 40 for the BRACETS A2 and A3 projections and 15 for KAUH-primary, reflecting the smaller patient-level sample. The projections were generated from the same pooled out-of-fold evaluation units used for the corresponding performance analyses and were not used for fitting, threshold selection, or statistical testing.

### 2.4 Statistical analysis

We quantified uncertainty using 1,000 bootstrap replicates. Resampling was performed at the patient level for KAUH-primary and at the subject-cluster level for BRACETS, thereby retaining all recordings from each sampled BRACETS subject. Two-sided 95% confidence intervals were defined by the 2.5th and 97.5th percentiles. For between-model comparisons, paired resampling preserved predictions from the same held-out evaluation units. The pooled KAUH-primary out-of-fold predictions were additionally used to estimate the calibration slope and intercept, classwise ECE, one-vs-rest classwise Brier scores, and ECE in the intermediate Obstructive-probability range p∈[0.20,0.80].

## 3 Results

### 3.1 Multi-class validation on BRACETS

BRACETS A2 and A3 were evaluated at the recording level above (28). The comparison included *AST* (31), *CLAP Linear Probe* (32), *OPERA Linear Probe*, *OPERA Adapter* (10), and *Evi-CARD* before and after query tuning. Table 2 reports the results. Figures 3a–f shows the corresponding visualizations.

**Figure 3:**
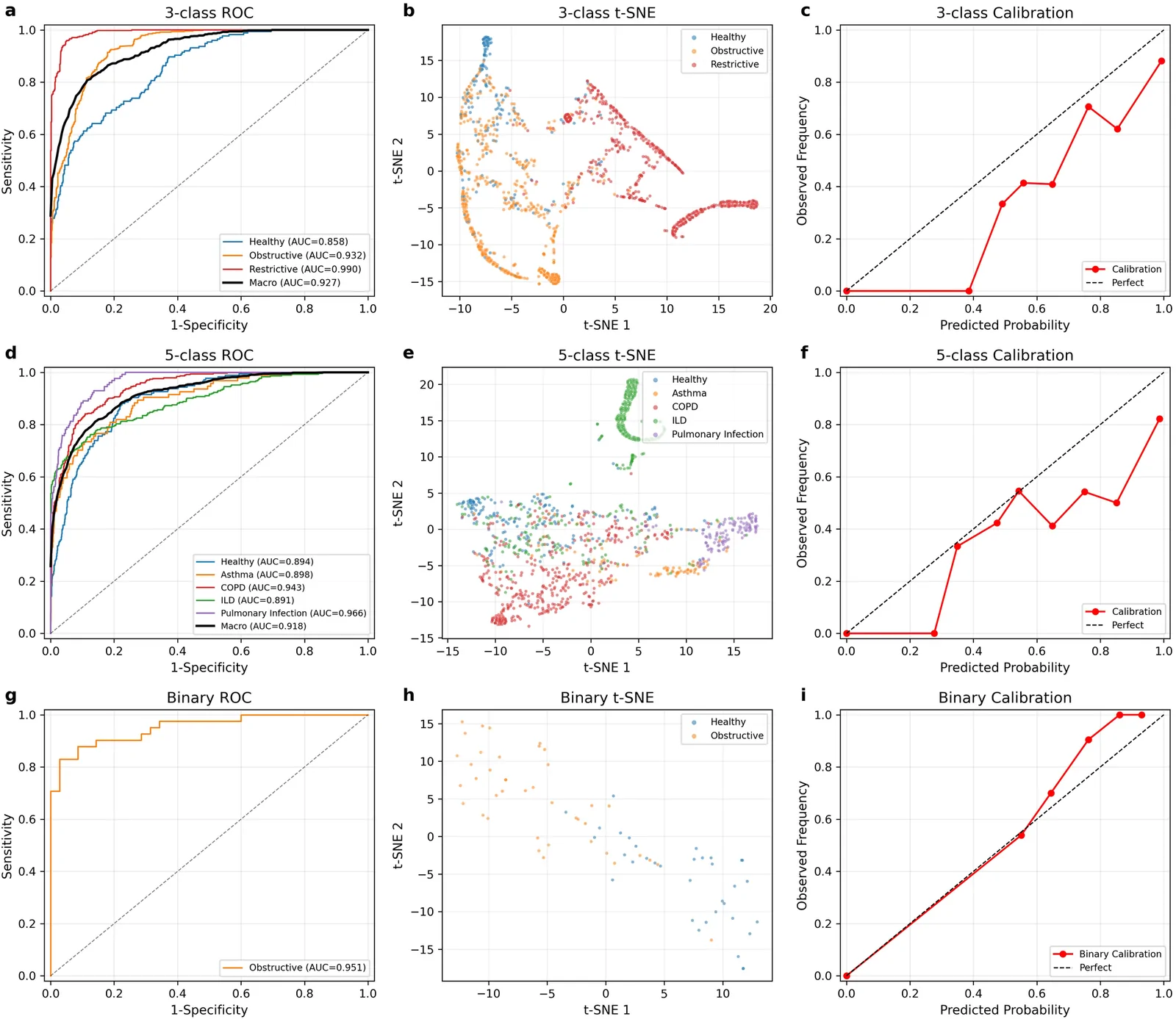
Performance visualization across BRACETS and KAUH-primary evaluation settings. **(a–c)** BRACETS A2 recording-level results for Healthy, Obstructive, and Restrictive categories. Panel **(b)** shows a comparatively localized Restrictive region and substantial Healthy–Obstructive overlap. **(d–f)** BRACETS A3 recording-level results for Healthy, COPD, Asthma, Interstitial Lung Disease (ILD), and Pulmonary Infection. Panel **(e)** shows localized ILD and Pulmonary Infection branches, whereas Healthy, Asthma, and COPD remain intermixed in the central region. **(g–i)** KAUH-primary patient-level five-fold validation for Healthy versus Obstructive classification, shown by the ROC curve, t-SNE projection, and calibration curve. In panel **(h)**, the two classes occupy partially separated regions with overlap near their interface rather than forming isolated clusters. The figure-generation script computes the A2 and A3 Macro AUROC legend values directly from the archived pooled probability vectors used for Table 2 and rounds them to three decimals. All t-SNE panels provide qualitative descriptions of projected probability structure and are not quantitative evidence of class separability or model performance.

For A2, *AST* and *CLAP Linear Probe* reached balanced accuracies of 63.4% and 67.2%, respectively. *OPERA Linear Probe* increased the A2 balanced accuracy to 69.3%, while *OPERA Adapter* reached 72.0%. The same ordering was observed under A3, where the corresponding balanced accuracies were 52.7%, 56.0%, 60.2%, and 63.3%. Across the baseline and *Evi-CARD* configurations, A3 balanced accuracy was 7.4–11.2 percentage points lower than A2. A3 retains separate COPD, Asthma, ILD, and Pulmonary Infection categories, whereas A2 uses the broader Obstructive and Restrictive groupings.

Before query tuning, *Evi-CARD* reached balanced accuracies of 74.7% on A2 and 67.3% on A3. After query tuning, the corresponding balanced accuracies increased to 77.8% and 70.2%, with pooled Macro AUROCs of 92.7% and 91.8%, respectively. Macro F1 values on A2 were 71.6% for *OPERA Adapter*, 74.3% for *Evi-CARD* before query tuning, and 77.5% after query tuning. On A3, the corresponding Macro F1 values were 62.9%, 66.5%, and 69.1%.

Calibration also improved across these configurations. On A2, ECE decreased from 15.1% for *OPERA Adapter* to 14.0% for *Evi-CARD* before query tuning and 13.1% after query tuning. The corresponding A3 values were 20.4%, 18.9%, and 18.0%. Brier scores were 0.33, 0.30, and 0.28 on A2 and 0.50, 0.47, and 0.45 on A3, respectively. Confusion matrices for the query-tuned configuration are shown in Figure 4.

**Figure 4:**
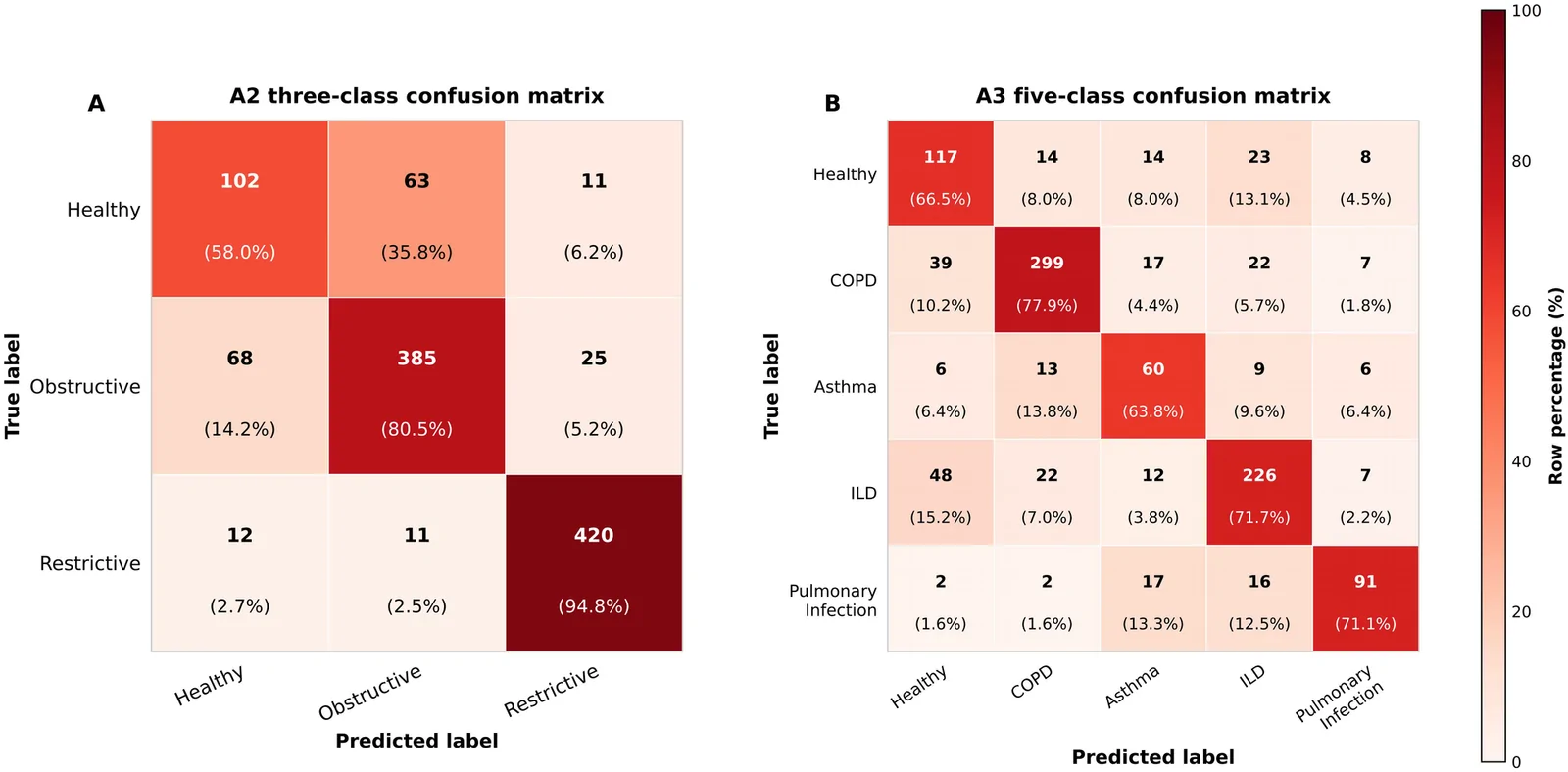
BRACETS recording-level confusion matrices for *Evi-CARD* with query tuning. **(A)** A2 three-class evaluation. **(B)** A3 five-class evaluation. Cell annotations show the exact aggregated integer counts and row percentages rounded to one decimal place. The pooled A2 and A3 balanced accuracies and class recalls in Table 2 are calculated directly from these integer counts.

### 3.2 Patient-level validation on KAUH-primary

KAUH-primary was evaluated as a Healthy vs. Obstructive classification task. The evidence pathway remained fixed, while a KAUH-specific binary disease-query layer was fitted within each training fold. Table 3 reports pooled patient-level out-of-fold point estimates on the first line and unweighted five-fold mean±standard deviation in parentheses. Figures 3g–i presents the same pooled out-of-fold predictions in the ROC curve, probability projection, and calibration curve.

Among the non-OPERA baselines, our MTL-MobileNet reimplementation achieved the highest AUROC at 89.1%, compared with 84.6% for *CLAP Linear Probe* and 86.3% for HeAR Linear Probe. *OPERA Linear Probe* reached an AUROC of 92.4%, a balanced accuracy of 85.8%, and an ECE of 9.8%. Fitting the OPERA adapter increased AUROC to 93.6% and balanced accuracy to 87.4%, while ECE decreased to 6.4%.

The Concept Bottleneck baseline reached an AUROC of 92.9% and a balanced accuracy of 86.0%. Its Brier score was 0.118 and its ECE was 7.1%, compared with 0.101 and 6.4% for *OPERA Adapter*. The Concept Bottleneck baseline had lower AUROC and balanced accuracy and higher Brier score and ECE than *OPERA Adapter*.

*Evi-CARD* reached an AUROC of 94.4%, an AUPRC of 93.4%, a balanced accuracy of 88.8%, and a Macro F1 of 88.0%. Its Brier score was 0.086 and its ECE was 5.3%. After KAUH query tuning, the pooled out-of-fold AUROC was 95.1%, balanced accuracy was 88.0%, and Macro F1 was 88.1%. The final Brier score and ECE were 0.083 and 5.1%, respectively. Sensitivity and specificity were calculated using the decision threshold selected within the corresponding training-validation partition.

The t-SNE projections in Figure 3 reveal local organization without complete class separation. In A2 (panel b), Restrictive samples occupy a comparatively localized region, whereas Healthy and Obstructive samples overlap over much of the remaining projection. In A3 (panel E), ILD and Pulmonary Infection form localized branches, while Healthy, Asthma, and COPD remain substantially intermixed in the central region. In KAUH-primary (panel h), Healthy and Obstructive patients are only partially separated, with a visible overlap at the class interface. These patterns help localize the remaining ambiguity: the finer A3 taxonomy produces the greatest interclass mixing, whereas the binary KAUH-primary task retains a clearer, though incomplete, boundary. The projections are descriptive and are not used as quantitative evidence of separability or predictive performance.

### 3.3 Bootstrap uncertainty and calibration analysis

Patient-level resampling on KAUH-primary placed the pooled final AUROC of 0.951 within a 95% CI of 0.9014–0.9875. The pooled threshold decisions corresponded to 37 of 41 Obstructive patients and 30 of 35 Healthy patients classified correctly, giving sensitivity 0.902, specificity 0.857, balanced accuracy 0.880 (95% CI, 0.8021–0.9492), and Macro F1 0.881 (0.8021–0.9472). The Brier-score interval was 0.0769–0.1352 around the manuscript point estimate of 0.083. These pooled values are also the headline point estimates in Table 3; the values in parentheses there remain explicitly labeled fold mean ± standard deviation and are not paired with the bootstrap intervals. Paired comparisons were retained for AUROC and Brier score. Relative to OPERA Adapter, the final configuration changed AUROC by +1.5 percentage points (95% CI, −1.6 to 4.7) and Brier score by −0.018 (−0.043 to 0.008); relative to Concept Bottleneck, the corresponding changes were +2.2 (−1.2 to 5.8) and −0.035 (−0.064 to −0.007). On the pooled point-estimate scale, the descriptive balanced-accuracy and Macro-F1 differences were +0.6 and +1.6 points vs. OPERA Adapter and +2.0 and +2.9 points vs. Concept Bottleneck. Earlier paired intervals for these threshold-dependent metrics are not attached to the corrected pooled differences because they were generated from superseded summary values. Thus, only the reduction in Brier score relative to Concept Bottleneck had a retained paired interval excluding zero; the KAUH-primary data do not resolve the remaining paired differences.

The subject-clustered BRACETS analysis retained the performance gap between the coarser A2 task and the more granular A3 task. For A2, the 95% confidence intervals were 0.9153–0.9381 for AUROC (0.927), 0.7543–0.7996 for balanced accuracy (0.778), 0.7517–0.7987 for Macro F1 (0.775), and 0.2476–0.3093 for Brier score (0.28). The corresponding A3 intervals were 0.9035–0.9308 for AUROC (0.918), 0.6666–0.7307 for balanced accuracy (0.702), 0.6542–0.7190 for Macro F1 (0.691), and 0.4141–0.4993 for Brier score (0.45). Unlike the KAUH-primary comparisons, all paired BRACETS intervals against OPERA Adapter excluded zero. On A2, *Evi-CARD* changed AUROC by +3.4 points (95% CI, 1.8–5.1), balanced accuracy by +5.8 (2.7–8.9), Macro F1 by +5.9 (2.5–9.2), and Brier score by −0.05 (−0.083 to −0.021). The respective A3 differences were +7.2 (4.7–9.8), +7.0 (2.8–11.3), +6.2 (1.9–10.6), and −0.05 (−0.091 to −0.014), indicating concordant gains in discrimination, class-balanced performance, and probabilistic accuracy under the present resampling protocol.

The classwise KAUH-primary analysis produced the same ECE for Healthy and Obstructive (11.78%), giving a mean of 11.78% (bootstrap 95% CI, 10.27%–19.37%). The one-vs-rest Brier score was likewise 0.083 for each class and in the classwise mean (0.0769–0.1352). These equal values follow from the binary formulation rather than from duplicated estimates: Healthy probabilities and labels are the complements of their Obstructive counterparts, which makes the two Brier components algebraically identical and the equal-width ECE bins mirror one another. The calibration slope was 2.204 (1.546–4.562), with an intercept of −0.279 (−1.816 to 0.523).

Among intermediate-probability predictions, 44 of 76 patients (57.9%) had an Obstructive probability in p∈[0.20,0.80]; ECE in this subset was 11.66% (9.11%–25.50%). This value should not be conflated with either the 5.1% global ECE, which bins maximum-class confidence against correctness for all patients, or the 11.78% classwise ECE, which bins one-vs-rest probabilities against indicator labels over the full probability range. The three summaries target different aspects and subsets of calibration; together, they show why the lower global ECE does not by itself characterize performance in the intermediate-probability region.

### 3.4 Module ablation analysis

Table 4 compares progressively constructed configurations using patient-level out-of-fold predictions. Adding the OPERA adapter increased AUROC from 92.4% to 93.6% and balanced accuracy from 85.8% to 87.4%. ECE decreased from 9.8% to 6.4%. The single-layer concept readout achieved lower discrimination and calibration, with an AUROC of 88.7%, a balanced accuracy of 80.4%, and an ECE of 13.6%.

**Table 4:** Ablation on KAUH-primary.

Adapter	MLQ	Dict.	Memory	Evid. wt.	CDQD	QT	AUROC (%)	BAcc (%)	Macro F1 (%)	ECE (%) ↓
×	×	×	×	×	×	×	92.4	85.8	84.2	9.8
✓	×	×	×	×	×	×	93.6	87.4	86.5	6.4
✓	SL	×	×	×	×	×	88.7	80.4	79.1	13.6
✓	✓	×	×	×	×	×	91.8	84.9	83.7	9.4
✓	✓	✓	×	×	×	×	93.1	86.6	85.9	6.9
✓	✓	✓	✓	×	×	×	96.4	84.1	82.9	12.1
✓	✓	✓	✓	✓	×	×	92.7	87.8	86.8	5.8
✓	✓	✓	✓	✓	✓	×	94.4	88.8	88.0	5.3
✓	✓	✓	✓	✓	✓	✓	95.1	88.0	88.1	5.1

MLQ, multi-layer query; Dict., explicit concept dictionary; Evid. wt., learned evidence weighting; QT, query tuning; SL, single-layer concept readout.

Learned evidence weights are model-internal gating variables and are not validated measures of recording quality, noise, fidelity, or annotator agreement. The rows form a progressive architecture analysis rather than a one-factor-at-a-time ablation. CDQD-absent rows use a linear classifier on the corresponding stage output.

The ablation trajectory was not monotonic across metrics. Adding the explicit concept dictionary to the multilayer concept-query configuration increased AUROC from 91.8% to 93.1% and reduced ECE to 6.9%. Residual memory without learned evidence weighting yielded the highest intermediate AUROC (96.4%) but poorer decision balance and calibration, with a balanced accuracy of 84.1% and an ECE of 12.1%. Introducing learned evidence weighting shifted this trade-off: balanced accuracy increased to 87.8% and ECE decreased to 5.8%, while AUROC decreased to 92.7%. Thus, improved ranking, class-balanced decisions, and calibration did not necessarily occur in the same configuration.

Adding *CDQD* increased balanced accuracy from 87.8% to 88.8% and reduced ECE from 5.8% to 5.3%. Query tuning then produced the final pooled configuration with 95.1% AUROC, 88.0% balanced accuracy, 88.1% Macro F1, and 5.1% ECE. Because several rows change more than one component and metric changes were not uniformly directional, differences between nonadjacent configurations should not be treated as isolated component effects.

Bootstrap uncertainty estimates reinforced this trade-off. Without learned weighting, residual memory achieved an AUROC of 96.4% (91.9%–99.1%) but a balanced accuracy of 84.1% (75.6%–91.6%), a Macro F1 of 82.9% (73.8%–90.4%), and an ECE of 12.1% (6.8%–19.3%). Learned weighting produced a more balanced profile: AUROC 92.7% (86.5%–97.4%), balanced accuracy 87.8% (79.5%–94.1%), Macro F1 86.8% (78.4%–93.2%), and ECE 5.8% (2.6%–10.9%). For the full configuration, the pooled bootstrap point estimates were 95.1% (90.1%–98.8%) AUROC, 88.0% (80.2%–94.9%) balanced accuracy, and 88.1% (80.2%–94.7%) Macro F1; its fold-summary ECE remained 5.1% (2.2%–9.7%). The pooled point estimates and fold summaries are reported separately to avoid treating two estimands as interchangeable.

Paired estimates identify which parts of this pattern were most stable. Adding learned evidence weighting changed AUROC by −3.7 points (95% CI, −7.1 to −0.5) and ECE by −6.3 (−11.2 to −1.7); both intervals excluded zero. The accompanying changes in balanced accuracy (+3.7, −1.8 to 9.0) and Macro F1 (+3.9, −1.3 to 9.4) were less precisely resolved. Comparing the full model with memory plus learned weighting gave paired changes of +2.4 in AUROC (−0.8 to 5.6) and −0.7 in ECE (−3.4 to 2.1); both intervals included zero. Under the corrected pooled estimand, the corresponding descriptive point differences were +0.2 points in balanced accuracy and +1.3 points in Macro F1. We do not attach the earlier paired BAcc and Macro-F1 intervals to these corrected pooled point estimates because those intervals were generated with the superseded 89.0%/88.4% summary values. Results across three random seeds showed the same qualitative profile. AUROC/balanced-accuracy/ECE were 96.2±0.9/84.3±1.7/12.0±1.5% without weighting, 92.9±1.1/87.6±1.4/5.9±0.8% with weighting, and 95.0±0.7/89.1±1.2/5.2±0.6% for the full configuration.

### 3.5 Concept-level validation

As shown in Figure 5, HF_Lung_V1, SPRSound, and ICBHI provided held-out concept-level evaluations under different acquisition and annotation conditions (8, 27, 29). The metrics in Table 5 assess recognition of wheeze, crackle, inspiration, and expiration, together with concept-level calibration.

**Figure 5:**
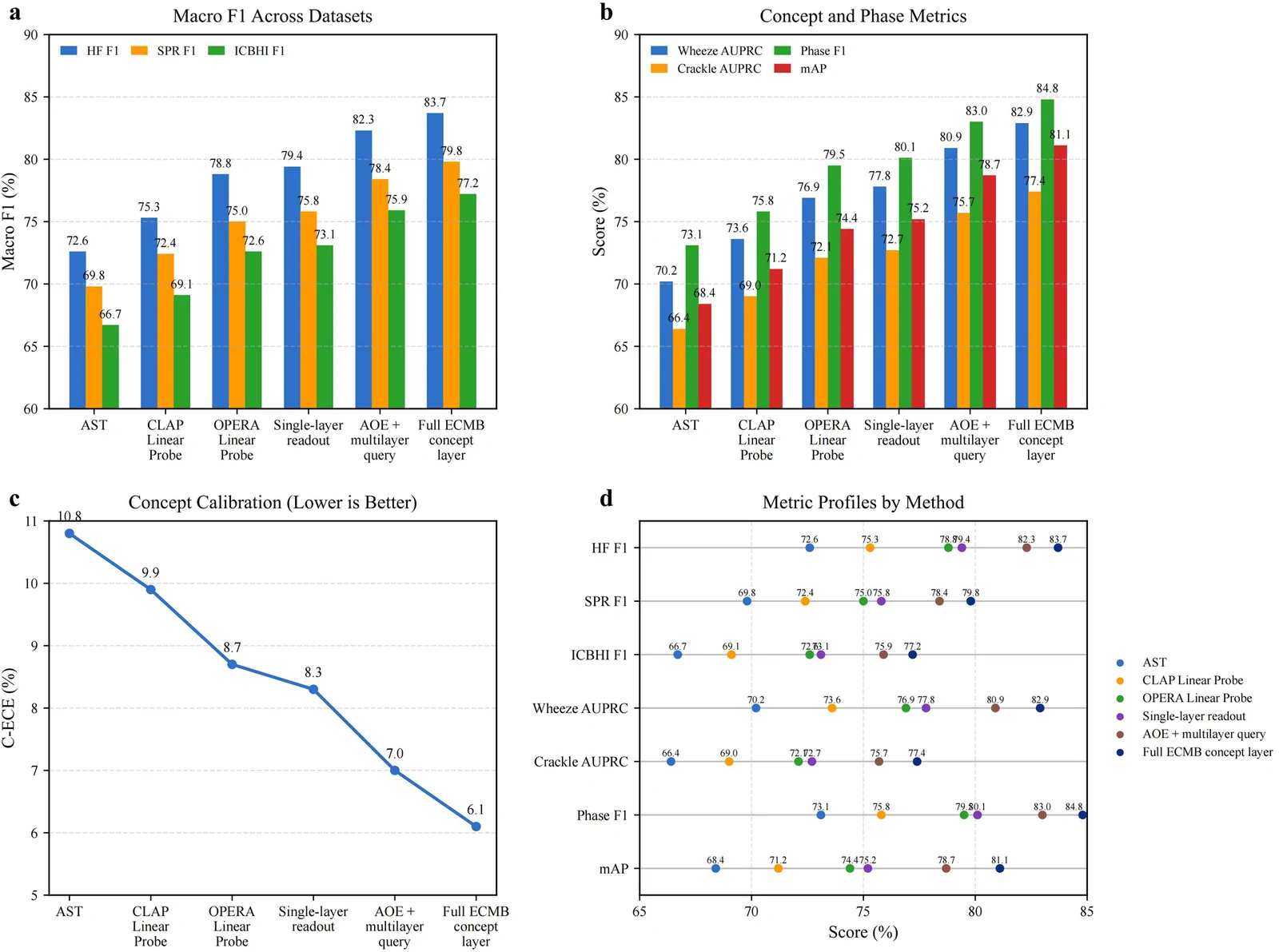
Concept-level metrics across respiratory sound datasets. **(a)** Macro F1 comparison across HF Lung V1, SPRSound, and ICBHI. **(b)** Concept- and phase-specific performance. **(c)** Concept-level calibration measured by C-ECE. **(d)** Metric profiles across methods.

**Table 5:** Concept-level validation results across HF_Lung_V1, SPRSound, and ICBHI.

Method	HF F1 (%)	SPR F1 (%)	ICBHI F1 (%)	Wheeze AUPRC (%)	Crackle AUPRC (%)	Phase F1 (%)	mAP (%)	C-ECE (%) ↓
AST	72.6 (72.5 ± 3.7)	69.8 (70.1 ± 2.4)	66.7 (66.8 ± 4.1)	70.2 (70.3 ± 4.5)	66.4 (66.7 ± 3.8)	73.1 (72.9 ± 2.2)	68.4 (68.1 ± 3.9)	10.8 (11.0 ± 2.1)
CLAP Linear Probe	75.3 (75.4 ± 3.1)	72.4 (72.6 ± 3.8)	69.1 (68.8 ± 3.3)	73.6 (73.9 ± 3.9)	69.0 (68.9 ± 4.2)	75.8 (75.6 ± 3.1)	71.2 (71.0 ± 3.5)	9.9 (9.7 ± 1.6)
OPERA Linear Probe	78.8 (78.5 ± 4.3)	75.0 (75.2 ± 3.0)	72.6 (72.3 ± 4.6)	76.9 (77.3 ± 4.1)	72.1 (72.3 ± 3.5)	79.5 (79.2 ± 2.9)	74.4 (74.7 ± 4.2)	8.7 (8.6 ± 1.9)
Single-layer readout	79.4 (79.2 ± 2.8)	75.8 (75.9 ± 3.5)	73.1 (72.9 ± 2.9)	77.8 (78.0 ± 3.4)	72.7 (72.4 ± 2.7)	80.1 (79.8 ± 2.5)	75.2 (75.3 ± 2.7)	8.3 (8.4 ± 1.4)
OPERA Adapter + MLQ	82.3 (82.7 ± 3.5)	78.4 (78.3 ± 2.7)	75.9 (75.4 ± 3.7)	80.9 (81.0 ± 3.1)	75.7 (76.0 ± 3.3)	83.0 (82.5 ± 2.1)	78.7 (78.4 ± 3.8)	7.0 (7.2 ± 1.7)
Full ECMB concept layer	83.7 (83.6 ± 2.9)	79.8 (79.9 ± 3.3)	77.2 (77.1 ± 2.5)	82.9 (83.0 ± 4.4)	77.4 (77.0 ± 2.8)	84.8 (85.1 ± 2.6)	81.1 (81.3 ± 3.1)	6.1 (6.4 ± 2.3)

HF denotes HF_Lung_V1; SPR denotes SPRSound; C-ECE denotes concept-level expected calibration error.

On HF_Lung_V1, *AST* reached a Macro F1 of 72.6%, while *CLAP Linear Probe* and *OPERA Linear Probe* reached 75.3% and 78.8%, respectively. The single-layer concept readout produced a Macro F1 of 79.4%. The adapter-augmented OPERA encoder with multilayer concept queries reached 82.3%, and the full *ECMB* concept layer reached 83.7%.

The same ordering was observed on SPRSound and ICBHI. The full *ECMB* concept layer reached Macro F1 values of 79.8% on SPRSound and 77.2% on ICBHI, compared with 78.4% and 75.9% for the multilayer-query configuration. For the pooled concept metrics, the full concept layer reached a wheeze AUPRC of 82.9%, a crackle AUPRC of 77.4%, a phase F1 of 84.8%, and an mAP of 81.1%.

Concept ECE decreased from 10.8% with *AST* to 8.7% with *OPERA Linear Probe*, 7.0% with the multilayer-query configuration, and 6.1% with the full *ECMB* concept layer. Relative to the multilayer-query configuration, the full concept layer increased mAP by 2.4 percentage points and reduced Concept ECE by 0.9 percentage points.

The incremental effect of the complete *ECMB* was similar across the three concept datasets: Macro F1 increased from 82.3% to 83.7% on HF_Lung_V1 (+1.4 percentage points), from 78.4% to 79.8% on SPRSound (+1.4), and from 75.9% to 77.2% on ICBHI (+1.3). Observing a comparable increment on SPRSound indicates that this pattern was not confined to datasets reported among OPERA’s pretraining resources. It remains a source-level sensitivity comparison, however, and does not establish external clinical generalization.

### 3.6 Residual-memory controllability analysis

Table 6 shows that concept-aligned evidence carried most of the predictive signal. Concept-only reasoning achieved 94.3% AUROC, 88.3% balanced accuracy, and 87.7% Macro F1, whereas residual-memory-only reasoning reached 90.6%, 84.0%, and 83.1%, respectively. Adding unrestricted memory to the concept pathway increased these point estimates to 95.6%, 89.7%, and 89.0%. Imposing the attention ceiling at ρ=0.35 yielded 95.1% AUROC, 88.0% balanced accuracy, and 88.1% Macro F1. This comparison supports a complementary role for residual memory while reiterating that the ceiling constrains attention allocation, not the maximum downstream logit contribution.

**Table 6:** Dynamically gated residual-memory attention analysis.

Setting	Concept-aligned	Dynamic memory	Memory ceiling	AUROC (%)	BAcc (%)	Macro F1 (%)
Concept-only	Yes	No	0	94.3 (94.1 ± 2.8)	88.3 (88.4 ± 3.3)	87.7 (87.5 ± 2.7)
Residual-memory-only	No	Yes	1.0	90.6 (90.7 ± 3.1)	84.0 (83.8 ± 2.4)	83.1 (82.9 ± 4.2)
Concept + unrestricted memory	Yes	Yes	Unlimited	95.6 (95.8 ± 2.5)	89.7 (89.9 ± 3.7)	89.0 (89.2 ± 3.5)
Concept + constrained memory	Yes	Yes	0.35	95.1 (95.0 ± 3.4)	88.0 (88.8 ± 2.9)	88.1 (88.5 ± 3.8)

The memory ceiling limits the maximum attention mass assigned to residual-memory positions; it does not strictly bound their logit-level contribution. For residual-memory-only reasoning, the ceiling is 1.0 because no concept-aligned evidence is available. In the mixed constrained configuration, ρ=0.35 limits residual-memory attention while preserving the concept-aligned pathway.

The paired AUROC analysis did not resolve a large independent benefit from this complementary pathway. Relative to concept-only reasoning, the constrained configuration changed AUROC by +0.8 percentage points (95% CI, −1.4 to 3.1). Under the corrected pooled estimand, its descriptive changes were −0.3 points in balanced accuracy and +0.4 points in Macro F1. The earlier paired intervals for these two threshold-dependent metrics are not attached to the corrected pooled values because they were generated with the superseded 89.0%/88.4% summary estimates. The AUROC interval included zero, supporting a modest complementary role rather than a statistically resolved, large performance gain.

## 4 Discussion

### 4.1 Protocol-specific respiratory sound classification

Respiratory acoustic foundation models provide useful representations for downstream classification (10), but the BRACETS results show that task definition remains consequential. *Evi-CARD* exceeded the OPERA linear-probe and adapter point estimates for balanced accuracy and Macro F1 on both protocols, with paired bootstrap intervals against OPERA Adapter excluding zero. Nevertheless, performance was consistently lower on A3, which distinguishes Asthma from COPD and ILD from Pulmonary Infection, than on A2, which merges these conditions into broader categories. Because label granularity, class distribution, and sample composition changed together, the present experiments cannot attribute the A2–A3 gap to any one factor.

Using separate disease-query layers allowed the same frozen evidence pathway to support A2, A3, and the patient-level Healthy vs. Obstructive task in KAUH-primary. This result concerns protocol adaptation, not cross-cohort generalization. In particular, most paired KAUH-primary intervals against the strongest baselines included zero, limiting the evidence to comparable or numerically improved performance within that cohort. Because BRACETS and KAUH-primary differ in cohort composition, labels, and evaluation units, their results constitute separate within-cohort experiments rather than a cross-dataset transfer or prospective validation study.

### 4.2 Explicit auscultation concept evidence

Concept-based learning makes named attributes part of the prediction pathway (21, 23), an approach also explored with clinically recognized attributes in dermatology (24, 25). Across HF_Lung_V1, SPRSound, and ICBHI, the complete *ECMB* layer improved concept Macro F1 and reduced concept ECE relative to the evaluated acoustic probes and simpler concept readouts. The incremental gain was also observed on SPRSound, which was not listed among the selected OPERA checkpoint’s pretraining resources; this is a source-level exposure sensitivity comparison, not an external-validation result. Overall, these findings support alignment of the intermediate streams with wheeze, crackle, inspiration, and expiration; they do not demonstrate semantic purity of every latent dimension. Stronger interpretability claims require direct concept interventions and independent clinician assessment.

### 4.3 Attention-constrained residual evidence

The residual pathway addresses the incompleteness of the four-concept vocabulary, but the controllability analysis places its contribution in perspective. Concept-only reasoning retained most of the final discrimination, and the retained paired AUROC interval comparing it with the attention-constrained configuration included zero. Residual memory is therefore best viewed as a modest complementary route for information outside the predefined concepts, rather than as the principal source of performance. Moreover, the ceiling constrains disease-query attention to residual positions without strictly bounding their causal contribution after value projection, and the learned slots have no assigned clinical meanings.

The four-stage training strategy reinforces this separation of roles: data partitioning precedes shared evidence learning, after which protocol-specific disease-head fitting and low-learning-rate refinement/calibration are performed without continuously altering the concept-aligned representation.

### 4.4 Learned evidence weighting

Learned evidence weighting altered the trade-off among ranking, decision balance, and calibration rather than improving every metric simultaneously. Applied to residual memory, it increased balanced accuracy and reduced ECE but decreased AUROC. The paired intervals resolved the AUROC reduction and ECE improvement, whereas the changes in balanced accuracy and Macro F1 remained uncertain. The evidence therefore supports a calibration-related trade-off rather than a uniform increase in discrimination. Because the weights were not supervised by acoustic-quality, noise, fidelity, or annotator-agreement labels, they remain model-internal gates and must not be interpreted as physical reliability estimates.

### 4.5 Limitations and overall interpretation

The study has several boundaries. First, BRACETS and KAUH-primary provide separate within-cohort cross-validation results; neither evaluates generalization across hospitals, stethoscope hardware, geographic populations, or prospective cohorts. Differences in acquisition conditions, recording sites, devices, filtering, environmental noise, populations, and annotation schemes further preclude direct comparison of performance across datasets. Second, KAUH-primary merges Asthma and COPD and therefore provides no evidence about subtype separation.

Third, aggregate metrics obscure clinically important error patterns. In BRACETS A2, Healthy recall was 58.0%, with 35.8% of Healthy recordings classified as Obstructive; such false-positive assignments could limit practical specificity. Persistent confusion among COPD, Asthma, ILD, and Pulmonary Infection in A3 likewise constrains subtype-level interpretation. Appropriate thresholds and the consequences of different errors must therefore be evaluated prospectively.

Fourth, crackle recognition remained weaker than wheeze recognition. Because crackles are brief and nonstationary, temporal down-sampling within an 8 s window may dilute their representation; shorter contexts or higher temporal resolution warrant evaluation. The current 8 s window and 4 s stride are also more consistent with segment-based or offline decision support than with instantaneous streaming auscultation. Real-time use would require incremental inference and dedicated assessment of computational latency, end-to-end response time, and stability during continuous acquisition.

Finally, concept alignment at the output level does not guarantee semantic purity of the underlying latent vectors. The residual pathway is not clinically interpretable, and the structured evidence fields have not been independently evaluated for clinical correctness or usefulness. Priority next steps are therefore device- and site-stratified testing, independent-cohort validation, direct concept interventions, prospective threshold evaluation, and blinded clinician review.

## 5 Conclusion

*Evi-CARD* structures frozen respiratory foundation-model representations into concept-aligned auscultation streams, learned evidence weights, and attention-constrained residual memory before protocol-specific classification. Across the evaluated protocols, it achieved balanced accuracies of 77.8% on BRACETS A2 and 70.2% on BRACETS A3, together with 95.1% AUROC and 5.1% ECE on KAUH-primary. The uncertainty analyses distinguish the strength of this evidence: paired improvements over OPERA Adapter were resolved on BRACETS, whereas most KAUH-primary comparisons were not, and the residual pathway showed only a modest complementary contribution. The model nevertheless makes named concept-associated fields available alongside disease probabilities. These within-cohort findings establish neither external generalization nor the semantic purity and clinical usefulness of those fields; independent-cohort and device-stratified testing, concept interventions, real-time latency evaluation, and blinded clinician review are required before deployment claims are warranted.

## Data Availability

Publicly available datasets were analyzed in this study. This data can be found here: the datasets used in this study are publicly available from their original repositories. BRACETS is available from Mendeley Data (DOI: 10.17632/f43c7snks5.1): https://data.mendeley.com/datasets/f43c7snks5/1. KAUH is available from Mendeley Data (DOI: 10.17632/jwyy9np4gv.3): https://data.mendeley.com/datasets/jwyy9np4gv/3. HF_Lung_V1 is available from GitLab: https://gitlab.com/techsupportHF/HF_Lung_V1. SPRSound is available from GitHub: SJTU-YONGFU-RESEARCH-GRP/SPRSound. The ICBHI 2017 Respiratory Sound Database is available from the official ICBHI repository: https://bhichallenge.med.auth.gr/ICBHI_2017_Challenge. RespiratoryDatabase@TR is available from Mendeley Data (DOI: 10.17632/p9z4h98s6j.2): https://data.mendeley.com/datasets/p9z4h98s6j/2.
